# Parameter sensitivity analysis for a stochastic model of mitochondrial apoptosis pathway

**DOI:** 10.1371/journal.pone.0198579

**Published:** 2018-06-18

**Authors:** Xianli Chen, Xiaoguang Li, Wei Zhao, Tiejun Li, Qi Ouyang

**Affiliations:** 1 The State Key Laboratory for Artificial Microstructures and Mesoscopic Physics, Department of Physics, Peking University, Beijing, China; 2 College of Mathematics and Compute Science, Hunan Normal University, Changsha, China; 3 Beijing Computational Science Research Center, Beijing, China; 4 Center for Quantitative Biology and Peking-Tsinghua Center for Life Sciences, Peking University, Beijing, China; 5 LMAM and School of Mathematical Sciences, Peking University, Beijing, China; University of Edinburgh, UNITED KINGDOM

## Abstract

Understanding how gene alterations induce oncogenesis plays an important role in cancer research and may be instructive for cancer prevention and treatment. We conducted a parameter sensitivity analysis to the mitochondrial apoptosis model. Both a nonlinear bifurcation analysis of the deterministic dynamics and energy barrier analysis of the corresponding stochastic models were performed. We found that the parameter sensitivity ranking according to the change of the bifurcation-point locations in deterministic models and the change of the barrier heights from a living to death state of the cell in stochastic models are highly correlated. For the model we considered, in combination with previous knowledge that the parameters significantly affecting the system’s bifurcation point are strongly associated with frequently mutated oncogenic genes, we conclude that the energy barrier height can be used as indicator of oncogenesis as well as bifurcation point. We provide a possible mechanism that may help elucidate the logic of cancer initiation from the view of stochastic dynamics and energy landscape. And we show the equivalence of energy barrier height and bifurcation-point location in determining the parameter sensitivity spectrum for the first time.

## 1. Introduction

It has been a consensus that cancer is a complex disease resulting from genomic alterations [1–2]. However, the mechanism of mutation-induced oncogenesis is not fully understood. In recent years, there are several interpretations have been proposed. Huang et al. [3] put forward the concept of ‘cancer attractors’ and thought that genetic aberrations would increase the region of cancer attractors or reduce the threshold of entering cancer basins. By constructing global potential landscape with simplified cellular networks, Li et al. [4] showed that gene mutations could trigger cell state transitions from normal to cancer states. Stites et al. [5] focused on the Ras functional module and demonstrated that gene mutations could alter the biochemical properties and further cause pathological changes. We presented a framework for mapping gene mutations to tumorigenesis [6–8], in which we analyzed nonlinear dynamic bifurcation behavior and investigated mutation enrichments in DNA damage induced apoptosis, Rb-related cell cycle and specific mitochondrial apoptosis pathways. We established that the location of the bifurcation point could be a threshold for cell cancerous changes [6–7], and subsequently verified the functional role of the bifurcation point with more precise relation between protein functional domain mutations and parameters [8].

The nonlinear dynamic bifurcation research in cancer-related networks is based solely on continuous and deterministic formalism. In reality, the biochemical reactions in cells are stochastic and discrete in nature. In particularly, when the number of reactant molecules is small, random fluctuations might be important, and cannot be negligible. What’s more, the stochastic effect will become apparent when the system is close to the bifurcation point, where the quasi-potential energy barrier becomes relatively low and the transitions become easier. This case happens even when the number of molecules is large. Thus, it is desirable to investigate the quantitative feature of the biological networks with stochastic dynamics and figure out what’s the indicators of oncogenesis in the corresponding stochastic systems, which is the aim of this paper.

**Fig 1:**
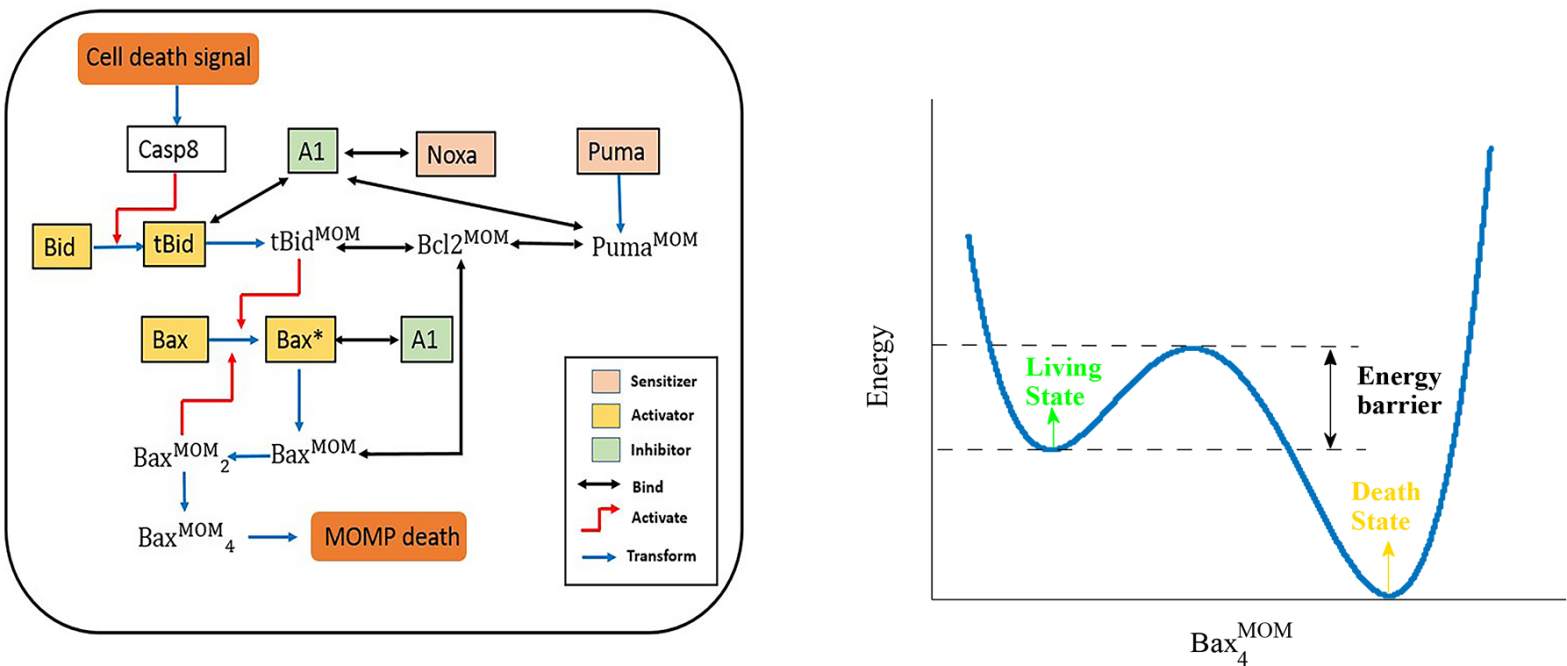
Schematic of the simplified mitochondrial apoptosis pathway and the energy landscape in terms of apoptosis effector. (a) Black lines indicate binding interactions, red lines represent activation and blue lines represent inhibition. Different functional proteins for apoptosis progression are colored, except for proteins with the MOM superscript which are on the mitochondrial outer membrane. Bcl-2 is representative of Bcl-2 Bcl-xl and Mcl proteins with anti-apoptosis properties. Bax is representative of Bax and Bak proteins. (b) The living and death states correspond to the two local minima of the potential function in terms of the apoptosis effector -Bax_4_^MOM^ variable. The energy barrier height characterizes the potential of the transition from living to death state.

Here, we used mitochondrial apoptosis dynamics to perform our study. From the perspective of the energy landscape (Fig 1B), the living and death states of the cell correspond to two steady states in the landscape, and the process of apoptosis corresponds to a dynamic path in which the system at steady state climbs an energy barrier and transits to another steady state. The height of this barrier indicates the likelihood of apoptosis. The higher the barrier is, the more difficult it for the cell to undergo apoptosis. Thus, it is more likely for the cell to become cancerous. By constructing the energy landscape of apoptosis dynamics, we can quantitatively study the potential of cancerization.

In this paper, we first determine the nonlinear bifurcation and parameter sensitivity analysis results for the deterministic dynamics of the mitochondrial apoptosis model. Then, we construct the corresponding stochastic model for the mitochondrial apoptosis pathway. By taking advantage of the quasi-potential landscape concept in probability theory [9,10] (see Sec. 2.3 and Sec. 1.7 in S1 File for a brief introduction), we compute the energy barrier height required for a cell to transit from a normal to a death state using the Geometric Minimum Action Method (GMAM) [9,10] and direct Stochastic Simulation Algorithm (SSA) [11]. We then study the changes in the barrier height in response to parameter variations and compare these results with those of the nonlinear bifurcation analysis. In addition, we perform Sobol sensitivity analysis which is a variance-based measurement and compare the results with the outcomes of the local analysis.

Through comparison and analysis, we find that the parameter sensitivity ranking according to the changes in bifurcation-point locations in deterministic models and the change in the barrier heights from living to death states of the cell in stochastic models are highly correlated. In particular, when the system is close to the bifurcation point, the parameter sensitivity patterns are almost identical. For the model that we considered here, our group previously showed that the parameters significantly affecting the system’s bifurcation point are strongly associated with frequently mutated oncogenes. Taken together, these results suggest that both the energy barrier height and bifurcation point position are indices of tumor formation.

## 2. Models and methods

### 2.1 Models

We focus on the mitochondrial apoptosis pathway, which is the most commonly deregulated form of cell death in cancer [12–14] and the cross-link of the intrinsic and extrinsic apoptosis pathways [15–17]. The mechanism of this pathway can be briefly summarized as follows: Caspase8 is an initiator caspase which can be activated in response to death stimulation, which originates from DNA replication stress, unfolded protein response or other causes. Activated Caspase8 further activates pro-apoptosis activators, such as Bid and BIM. The accumulation of activators will give rise to the oligomerization of the effectors Bax and Bak which results in pores in the mitochondrial membrane. The pores then trigger mitochondrial outer membrane permeabilization (MOMP). Subsequently intermembrane space proteins such as cytochrome c and Smac are released to the cytoplasm and eventually lead to downstream cascades and cell death [18–20]. We take advantage of the mitochondrial apoptosis model of Zhao et al [8] which conformed to the biological facts we have known. The entire regulation network is shown in Fig 1A. Proteins with redundant functions are compressed into one representative reactant. For example, Bax and Bak are the major effectors of apoptosis, either Bax or Bak alone is sufficient to form oligomer and then lead to MOMP, therefore, we used Bax as the representative. The ODEs in this network can be found in SI. We denote it by

$$\dot{\vec{x}}=b(\vec{x})$$ (1)

for short. $\vec{x}$ represent the reactants. The dynamic behavior of the system is governed by the function $b(\vec{x})$, which is derived according to the law of mass action.

In the progress of mitochondrial apoptosis, MOMP acts as a defining event that irreversibly commits cells to death. Consequently, for simplicity, cell MOMP is considered a death state without accounting for post-MOMP regulation. The concentration of Bax_4_^MOM^ is the output variable, and the Caspase8 concentration is the input variable. In this continuous-state deterministic dynamics, the system has two stable fixed points and one saddle point in the parameter region that we consider, which gives rise to a bistability character.

### 2.2 Stochastic model and simulation of mitochondrial apoptosis

Starting from the deterministic model described above, we now discuss the stochastic setup of the system. We model the mitochondrial apoptosis dynamics as a Markov jump process. We denote the state of the system by a vector *X* = (*X*_1_,*X*_2_,…,*X*_20_), whose *i-*th component represents the number of molecules for the *i-*th species in ODEs (1). Each reaction in Fig 1 can be described by a propensity function *a*_*j*_(*X*) and a state change vector *ν*_*j*_. The system volume size *V* plays an important role in describing the propensity function. There are three types of reactions in this system. The first type is when a certain molecule is added to the system from outside with a constant rate, such as $\phi \overset{k_{5}}{\to }tBid$. In this case, the propensity function is *a*(*X*) = *Vk*_5_. The second type is the involvement of only one species in the reaction, such as $Bid\overset{Cas8\;k_{1}}{\to }tBid$. The propensity function of this reaction is *a*(*X*) = *cas*8*k*_1_*X*_1_. For the last type of reaction, two types of molecules (including two of the same molecule) are involved, such as ${Bax}^{MOM}+{Bax}^{MOM}\overset{k_{12}}{\to }{Bax}_{2}^{MOM}$. The propensity function of this reaction is *a*(*X*) = *V*^−1^*k*_12_*X*_13_(*X*_13_ − 1).

State change vector characterizes the change of system state if a reaction fires. For example, in the $Bid\overset{Cas8\;k_{1}}{\to }tBid$ reaction, the state change vector is *ν* = (−1,1,0,…,0), which means that the Bid number will be decreased by 1 step reaction, and the tBid number will be increased by 1 by one step reaction. Once fired, the state of the system would be updated from *X* to *X* + *ν*. All other reactions can be described similarly. There are 53 total reactions in this system. Deterministic Eq (1) is the limit of the stochastic process *X*/*V* as *V* goes to infinity [21].

With this setup, Gillespie’s SSA algorithm can be used to directly simulate the system. To describe the event of apoptosis, we choose Bax_4_^MOM^ as a MOMP marker. In a single simulation, the death state corresponds to the number of Bax_4_^MOM^ larger than the threshold. Once the number of Bax_4_^MOM^ exceeds this threshold, the cell dies. The corresponding simulation time is called the first passage time (FPT). The oncogenic potential can be estimated as the mean first passage times (MFPT) from normal state to death state using Monte Carlo simulations.

### 2.3 Energy landscape and large deviation theory

To quantitatively understand mitochondrial apoptosis, we construct the energy landscape for apoptosis dynamics. For a general deterministic dynamical system, there is typically no natural potential landscape. In recent studies, Li et al. [9–10] showed that the quasi-potential in large deviation theory [22–23] is considered as one mathematical realization of the energy landscape.

The quasi-potential landscape is defined as follows. For any stable steady state *x*_0_ of Eq (1), the local quasi-potential *S*(*x*;*x*_0_) with respect to *x*_0_ is defined as the minimization of an action function

$$S(x;x_{0})={\inf\;\inf}_{T>0\;\varphi (0)=x_{0},\;\varphi (T)=x}\int_{0}^{T}L(\varphi ,\dot{\varphi })dt,$$ (2)

where *φ*(*t*) is a continuous path connecting *x*_0_ and *x*[24]. The function *L*(*x*,*y*), which can be considered as the Lagrangian, is the Legendre transform of a Hamiltonian *H*(*x*,*p*). Namely,

$$L(x,y)=\sup_{p}\left\{p∙y-H(x,p\right)\}.$$ (3)

For the Markov jump process described in Sec.2.2, the Hamiltonian has the form

$$H(x,p)=\sum_{j}a_{j}(x)(e^{p∙\nu_{j}}-1),$$ (4)

while *L*(*x*,*y*) does not have a closed form, where the summation is taken over all reactions. Based on the local quasi-potentials constructed from different stable steady states, the global quasi-potential *S*(*x*) is a proper combination of all local versions. One may refer to [25] for technical details.

The local quasi-potential *S*(*x*;*x*_0_) characterizes the difficulty of transition from *x*_0_ to *x*. Indeed, if we denote the FPT from *x*_0_ to *x* by *τ*, the large deviation theory tells us [21] that

$$\epsilon \;\log\;E\tau \to S(x)-S(x_{0})$$ (5)

when ϵ → 0, where **E***τ* is the mean first passage time. Thus, we can use the barrier height of the quasi-potential landscape to study the oncogenic potential quantitatively. The higher this barrier is, the more difficult it is for the cell to undergo apoptosis and the easier it is for a normal cell to change into a cancer cell. Let us denote the live state as the lower Bax_4_^M OM^ level by *x*_*low*_, and the death state as the higher Bax_4_^M OM^ level by *x*_*high*_. The minimum action *S* for the transition from *x*_*low*_ to *x*_*high*_ can be computed efficiently using the GMAM algorithm [11, 24]. We then utilize the obtained barrier heights to study the sensitivity of the oncogenic potential with respect to different parameter choices. The code for computing the barrier height with GMAM algorithm can be downloaded from the website (http://dsec.pku.edu.cn/~tieli/code/Apoptosis-Code.zip).

Notably, if we select *x*_0_ = *x*_*low*_ and *x* = *x*_*high*_, then the mean time **E***τ* is the mean transition time from *x*_*low*_ to *x*_*high*_. This time may be slightly different from the MFPT defined in Sec. 2.2. If we select a threshold less than *x*_*high*_, i.e. $x_{high}\in \{{Bax}_{4}^{MOM}>threshold\}$, then **E***τ* always overestimates MFPT in SSA simulation. Fortunately, when the volume size *V* is sufficiently large, the transition from *x*_*low*_ to *x*_*high*_ can be rare. Most of the transition time is spent climbing up the energy barrier. Once the system climbs over the barrier, it rapidly relaxes to *x*_*high*_. In such a case, both MFPT in the SSA simulation and **E***τ* are approximately equal to the time required to climb the energy barrier. Therefore, in practice, MFPT ≈ **E***τ*. With this approximation, MFPT also quantifies the energy barrier height, depicted as Arrhenius’ law. Thus, SSA can also be used to estimate the energy barrier height.

## 3. Results

### 3.1 The bifurcation and parameter sensitivity analysis of the deterministic model

**Fig 2:**
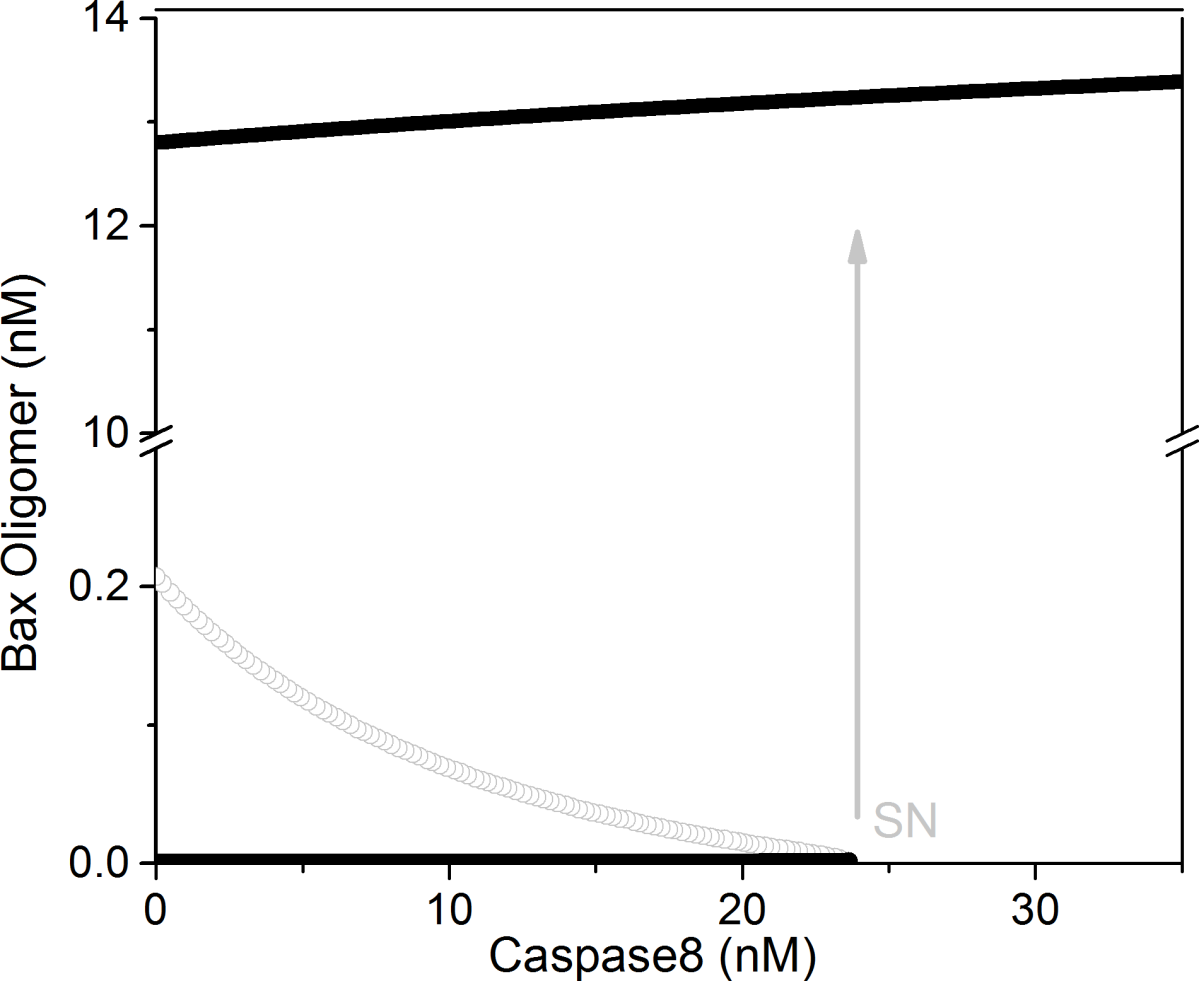
Caspase8 is taken as the control parameter, and the Bax_4_^MOM^ concentration is used as the output. SN indicates saddle-node. Black lines represent stable steady state, and gray line indicates an unstable steady state with wild-type (unchanged) parameters.

We take the concentration of Bax_4_^M OM^ as the output and the Caspase8 concentration as the input to develop a bifurcation diagram (as shown in Fig 2) of the deterministic model. Under the biological meaningful conditions, the system has bistable characteristics. The low stable steady state branch indicates the cell living state and the high stable steady state represents the death state. The location of the saddle-node (SN) bifurcation point is Cas8 = 23.59nM which is in the concentration range of Caspase8 to activate downstream effector caspases [26].

Here, we use two different methods, local single-parameter analysis and variance-based Sobol analysis [27–28], to obtain a parameter sensitivity spectrum. Local sensitivity analysis is a classical method that is used to examine the impact of small perturbations of parameters on model outputs, and Sobol sensitivity analysis is a global sensitivity analysis method that is used to study how large variations of parameters affect the outputs [29]. The local sensitivity analysis is typically efficient in computer time but may be inadequate for nonlinear models compared with the global sensitivity analysis methods [30].

**Fig 3:**
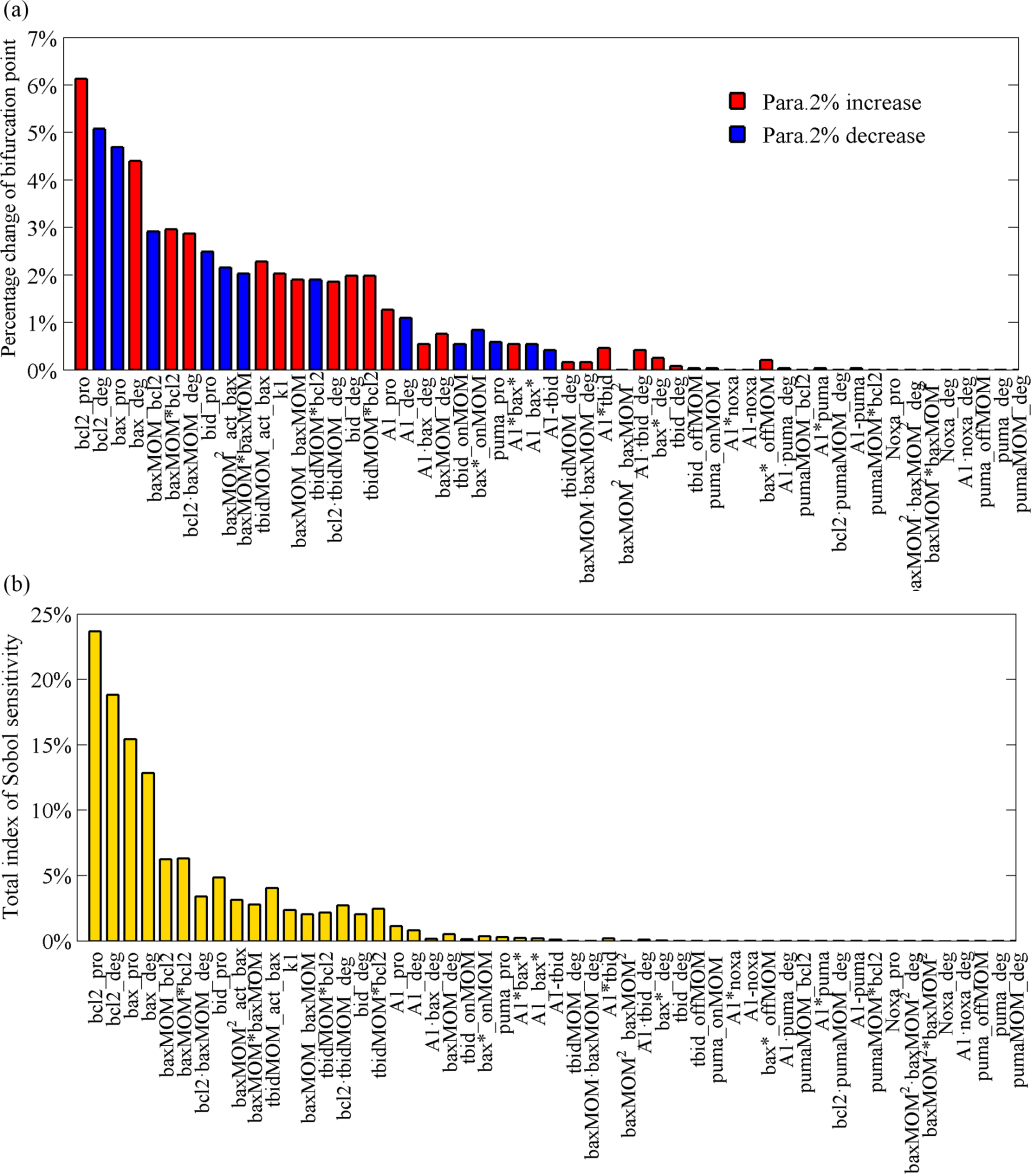
Parameter sensitivity spectrum in deterministic simulation. (a) A 2% decrease or increase in each parameter induced a percentage change of the bifurcation point SN with local sensitivity analysis method. Horizontal coordinates represent parameters of the model, and vertical coordinates represent percent changes of bifurcation-point location in response to the corresponding changes in the parameters. Details of the correspondence between parameters and representative interactions are shown in Supporting Information. Asterisk, association; deg, degradation rate; onMOM/offMOM, membrane translocation and membrane separation; pro, production rate; minus, dissociation. (b) Total effect index of each parameter calculated by using Sobol sensitivity methods.

Fig 3A presents the parameter sensitivity spectrum calculated using single-parameter sensitivity analysis with a 2% change of each parameter. Here, we mainly focus on the parameter changes that can increase cell oncogenic potential. A right shift of the bifurcation point suggests an increasing oncogenic potential [12], that is, moving the bifurcation point from left to right will make it difficult for cells’ death and be advantageous to the carcinogenic process. Therefore, the direction of parameter perturbation (increasing or decreasing) that leads to a right shift of the critical point is chosen. We also alter the perturbation size of the parameters (Fig A, B and C in S1 File) and find similar patterns of sensitivity as in Fig 3A.

The variance-based Sobol sensitivity analysis makes no assumptions about the relations between models’ inputs and outputs; it is a way to obtain global information on parameter sensitivity. In this calculation, we randomly generate parameters in a large parameter space using Sobol sequences and ten thousand sets of parameters that can render bistable behavior. Then, we use standard Sobol calculation method to get parameter sensitivity spectrum, as shown in Fig 3B. Here, we focus mainly on the total effect index, which quantifies the overall effects of a parameter. The principle theory of the Sobol sensitivity analysis are shown in SI. Spearman’s correlation of two different sensitivity analysis methods is 0.981, with a significance level less than 0.001.

### 3.2 Parameter sensitivity in the SSA simulation

**Fig 4:**
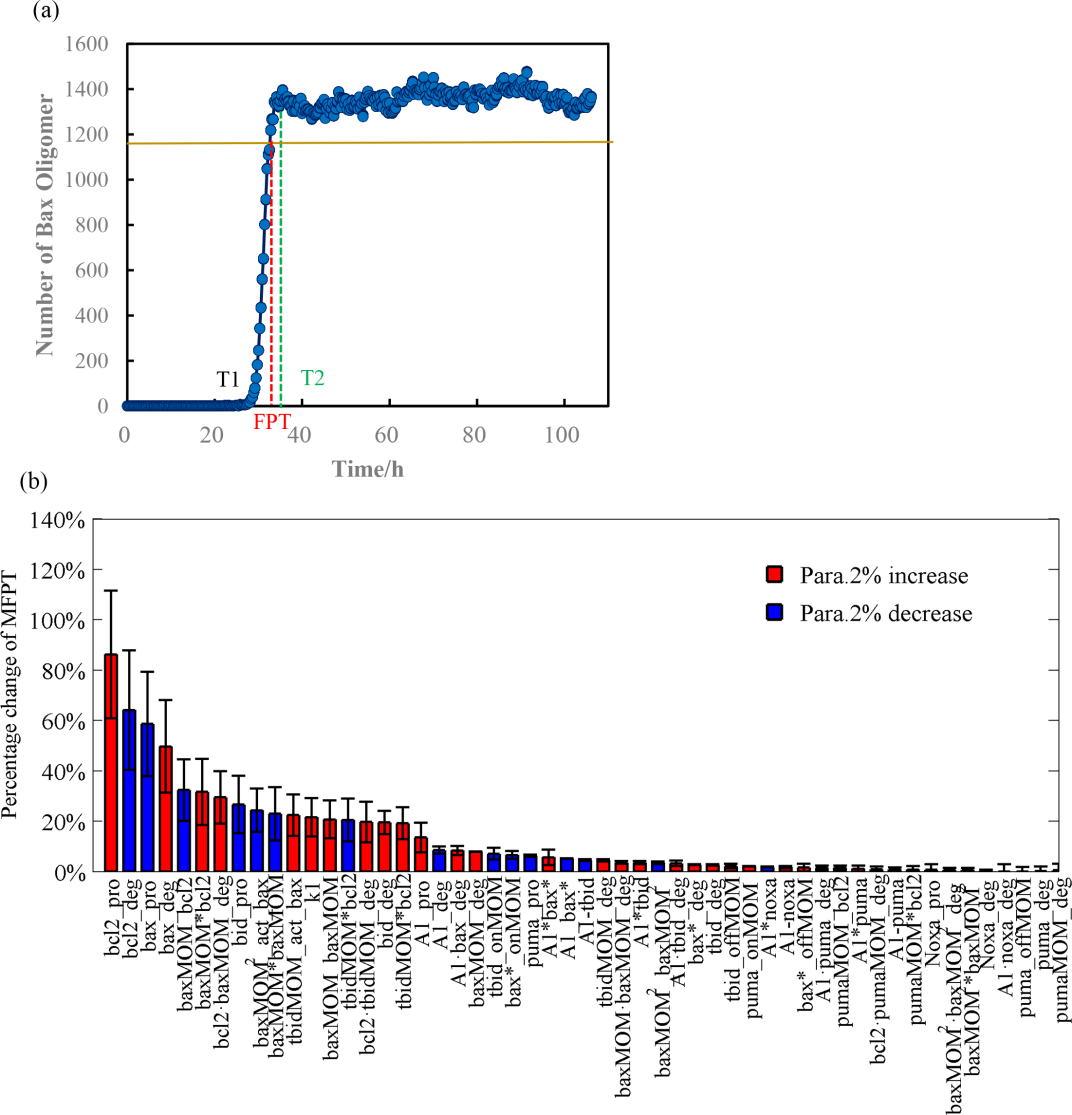
Dynamic trajectory and parameter sensitivity ranking in the SSA simulation. (a) Horizontal coordinates represent time; vertical coordinates represent the Bax_4_^MOM^ number. T1 indicates the time point at which the system starts to transform to a high state, T2 represents the time at which the first mean value of high steady state is reached, and FPT is a value between T1 and T2, and indicating the time when Bax_4_^MOM^ number first reaches 80% of mean value of high state. (b) Parameter sensitivity analysis in Gillespie stochastic simulations. Red histograms illustrate changes of MFPT in response to a 2% increase of the parameters. Blue histograms represent changes of MFPT in response to a 2% decrease of the parameters. Dark bars are shown the coefficient of variance. Increasing and decreasing in parameters are selected so the MFPT becomes longer.

As shown in Sec. 2.2, the event of MOMP is indicated as the Bax_4_^MOM^ number exceeding the threshold. In our simulation, first passage-time (FPT) is identified as the time when the Bax_4_^MOM^ number first reaches 80% of mean value of high state. Other thresholds like 50% and 90% of mean value of high state that characterize the change of low to high steady states are also used. There is almost no difference in sensitivity spectra using different thresholds (Fig D and E in S1 File). Fig 4A shows the dynamic changes of the Bax_4_^MOM^ number. The yellow horizontal line represents the threshold of counting the FPT. The horizontal coordinate of the red dashed line corresponds to FPT. The horizontal coordinate of the green dashed line represents the time required to reach a mean high steady state value. Considering a volume size of V = 100 *μm*^3^ Cas8 = 23 nM, the other parameters are the same as those in the deterministic model. Other volume sizes can also be chosen, the spectrum didn’t change much (Fig F and G in S1 File). We see that apoptosis also be activated without triggering the bifurcation with high levels of Cas8, as an important property obtained from our stochastic model. Ten thousand dynamic change trajectories with the same condition are obtained for statistical purposes. We calculate the mean first passage-time (MFPT) in response to a 2% change of each parameter. From this perspective, a longer MFPT indicates an enlarged oncogenic potential. Therefore, the direction of each parameter perturbation (increase or decrease) is chosen to make the MFPT longer. The parameter sensitivity ranked according to the influence that each parameter has on MFPT is shown in Fig 4B. The red and blue histograms illustrate the changes of MFPT in response to a 2% increase and decrease of the parameters, respectively.

### 3.3 Parameter sensitivity of energy barrier height

**Fig 5:**
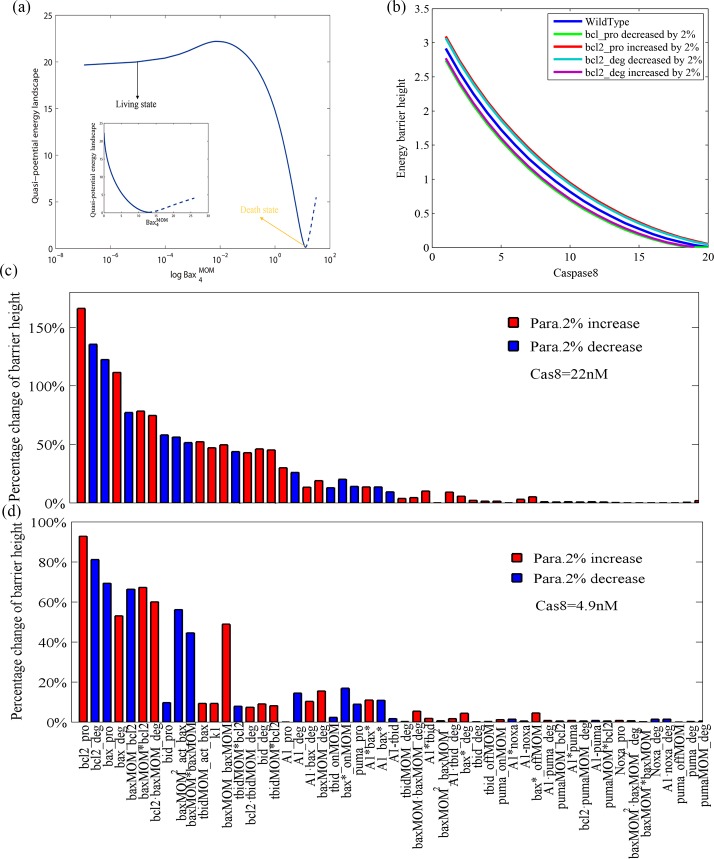
Parameter sensitivity spectrum of barrier height based on GMAM. (a) The quasi-potential energy landscape in terms of log Bax_4_^MOM^ when Cas8 = 4.9 nM. The small figure insert shows the quasi-potential energy landscape in terms of Bax_4_^MOM^. The two figures describe the same thing but with different abscissa variables (log Bax_4_^MOM^ and Bax_4_^MOM^). As shown in the right panel of Fig 1, the living and death states correspond to two local minima of the potential function in terms of the log Bax_4_^MOM^ or Bax_4_^MOM^. (b) Cas8 changes from 4 to 23 nM. The bifurcation point is Cas8 = 23.59 nM. Parameter bcl2_pro in the legend stands for production rate of Bcl2 and bcl2_deg represents degradation rate of Bcl2 that are the two most sensitive parameters. Blue, wild type parameters; green, bcl2_pro decreased by 2%; red, bcl2_pro increased by 2%; cyan, bcl2_deg decreased by 2%; and purple, bcl2_deg increased by 2%. (c) Parameter sensitivity spectrum of the quasi-potential barrier height when Cas8 = 22 nM, which is close to the bifurcation -point location of the corresponding deterministic dynamics. A 2% decrease or increase of each parameter induces the percentage change of energy barrier height according to the local sensitivity analysis method. (d) Parameter sensitivity spectrum of the quasi-potential barrier height when Cas8 = 4.9 nM, which is relatively far from the bifurcation point. A 2% decrease or increase of each parameter induced the percentage change of energy barrier height according to the local sensitivity analysis method.

Based on the GMAM method described in Sec. 2.3, we can compute the energy barrier of the quasi-potential energy landscape using the same parameter setup. Fig 5A shows the results of the quasi-potential energy landscape computation in terms of log Bax_4_^MOM^ when Cas8 = 4.9nM. The living and death states correspond to two local minima of the potential function. The result that the local landscape of death state is much deeper than that of living state is consistent with biological facts. The quasi-potential energy landscape in terms of Bax_4_^MOM^ when Cas8 = 22 nM is also shown (Fig H in S1 File). In Fig 5B, the energy barrier changes with Caspase8 input. Indeed, when Caspase8 increases to the bifurcation point, the energy needed to exit the normal state decreases until reaching 0. When Cas8 is far from bifurcation point, the transition from normal state to death state is rare. Direct SSA simulation cannot generate a reliable statistic of MFPT in a reasonable time. Luckily, the energy barrier can still be calculated. As shown in Sec. 3.1 and Sec. 3.2, we change each parameter by 2% and observe the changes in the barrier height. Fig 5C and 5D show the results.

The rank of sensitivity when Cas8 is near the bifurcation point (Fig 5C) coincides quite with the SSA simulation and ODEs analysis. The Spearman’s correlation between sensitivity of barrier height and MFPT in SSA is 0.973, with a significance level less than 0.001. Spearman’s correlation between the sensitivity of the barrier height and bifurcation point in ODEs is 0.981, with a significance level less than 0.001. Fig 5D shows the sensitivity of the barrier height change in response to the change in each parameter when Cas8 = 4.9 nM. One may find that the barrier height is more than 50 times larger than that of case Cas8 = 22 nM (shown in Fig 5B). The rank of sensitivity when Cas8 is relatively far from the bifurcation point shows slightly different from the results when Cas8 is close to the bifurcation point. This difference is mainly between parameters k5 (production rate of Bid) and kf12 (dissociation rate of membrane- binding Bax). This distinction suggests that the relative importance of the two parameters is different in low Cas8 and high Cas8 cells. The barrier height of our model can reflect the potential of cancer initiation. Thus, in cells with high Cas8 concentrations, the oncogenic potential of Bax^MOM^ dimer mutation is lower than that for Bid. Inversely, the oncogenic potential of Bax^MOM^ dimer mutation is higher than for Bid in low Cas8 cells. Spearman’s correlation between these two results shown in Fig 5C and 5D is 0.826, suggesting that the rank correlation of the two is quite good. The consistency of the rank when Cas8 = 4.9 nM and Cas8 = 22 nM suggests that the sensitivity of this parameter reveals the intrinsic property of the mitochondrial apoptosis dynamics, regardless of Cas8 input. Spearman’s rank correlations of parameter sensitivity with different methods are shown in Supporting Information (Table B in S1 File). By comparing the rank correlations, we can see that the parameter sensitivity spectrum according to bifurcation point position is similar to the spectrum obtained from the energy barrier analysis near bifurcation point.

## 4. Conclusion

For a bistable system, people may intuitively think that when the location of bifurcation point shifts, the energy landscape changes. However, the specific quantitative changes have scarcely been studied, particularly in complex biological systems. Using mitochondrial apoptosis as an example, we specifically compared the changes of bifurcation point and energy barrier height. We successfully calculated MFPT and energy barrier height using the SSA and GMAM algorithms. The resulting stochastic model provides the transition from different basins of attraction of metastable states, which cannot be described from the deterministic model. We observe activation of apoptosis without high levels of Cas8 triggering bifurcation from the stochastic model. Additionally, when a parameter change causes a shift of the bifurcation point, there is a corresponding change of the energy barrier height and vice versa. The higher the barrier height is, the more difficult it is for a cell to evolve to death, indicating increasing oncogenesis potential. A right shift of the bifurcation point also implies higher oncogenesis potential. These results confirmed the consistency between them.

We rank the parameters according to their influence on MFPT and energy barrier. Compared with the results of parameter ranking obtained from deterministic dynamic studies, we find a high consistence of parameter sort orders. The ‘equivalence’ of bifurcation point and energy barrier height in determining parameter sensitivity spectrum is also shown in Schlögl model which is a simple tri-molecular reaction model that can give bistability. The corresponding analysis process can be found in the Supporting Information. From sensitivity analysis shown above, we find that the production and degradation rates of Bcl-2 and Bax (bcl2_pro, bcl2_deg, bax_pro, and bax_deg) are the most sensitive, thus illustrating the crucial role of these two proteins. The increase of bcl2_pro, bax_deg and the decrease of bcl2_deg, bax_pro correspond to higher oncogenesis potential. This result is consistent with those of previous studies [31,32]. For example, chromosomal translocation-induced Bcl-2 overexpression and other amplification cases have been observed in many cancer types [33–34], so it is not surprising that the parameter describing Bcl-2 production stands out as the most sensitive one. Similarly, the frameshift mutations in a (G)8 mononucleotide tract of the Bax gene, which lead to synthesis of a truncated protein and reduced expression, commonly occur in colon and gastric cancers [35], suggesting that Bax inactivation during tumorigenesis may facilitate tumor progression by enhancing escape from apoptosis.

For the model we considered here, we have already shown that the parameters significantly affecting the system’s bifurcation point are closely correlated with high frequency mutations [8]. Thus, the bifurcation-point location can be considered as indicator of the potential of cell to become cancerous. Taken together, we conclude that energy barrier height can also be regarded as an indicator of oncogenesis.

In the mitochondrial apoptosis pathway, right shifts of bifurcation point render higher oncogenic potential. Consequently, a far-more-right position of the bifurcation point may render a normal cell cancerous. Pre-treatment of mitochondria that moves a cell near the threshold of apoptosis could be useful as an anti-cancer strategy.

## Supporting information

S1 File Supporting information. (PDF)
