# Supplementary material for: Parameter sensitivity analysis for a stochastic model of mitochondrial apoptosis pathway
Source: PLoS One. 2018 Jun 18;13(6):e0198579. doi: 10.1371/journal.pone.0198579 (PMC6005494; doi:10.1371/journal.pone.0198579)
Supplement: S1 File — (PDF) [file pone.0198579.s001.pdf]

Supporting Information

Xianli Chen[1 ☯], Xiaoguang Li [2 ☯], Wei Zhao[4], Tiejun Li [3*], Qi Ouyang [1,4*]

1.1 Ordinary differential equations of Mitochondrial apoptotic pathway dynamics

Eqs. (1) - (20) are ODEs that are translated according to the regulatory network of Fig. 1. They describe the dynamics of the mitochondrial apoptotic pathway in response to death signal. Caspase8 (C8) is the control parameter.

$$(1)\quad \frac{d[Bid]}{dt} = -C8 \cdot k_1[Bid] + k_5 \;\; - k_{f5}[Bid]$$

$$(2)\quad \frac{d[tBid]}{dt} = C8 \cdot k_1[Bid] - k_{tBidon}[tBid] + k_{tBidoff}[tBid^{MOM}] - k_{dtBid} \cdot [tBid] - k_{26}[A1][tBid] + k_{f26}[A1 \cdot tBid]$$

$$(3)\quad \frac{d[Bax]}{dt} = k_6 - k_{f6}[Bax] - k_3[tBid^{MOM}][Bax] - k_{16}[Bax^{MOM} \cdot Bax^{MOM}][Bax]$$

$$(4)\quad \frac{d[Bax^*]}{dt} = k_3[tBid^{MOM}][Bax] + k_{16}[Bax^{MOM} \cdot Bax^{MOM}][Bax] - k_{Baxon}[Bax^*] + k_{Baxoff}[Bax^{MOM}] - k_{dBax}[Bax^*] + k_{f24}[A1 \cdot Bax^*] - k_{24}[A1][Bax^*]$$

$$(5)\quad \frac{d[Bcl2]}{dt} = k_7 - k_{f7}[Bcl2] + k_{f2}[tBid^{MOM} \cdot Bcl2] - k_2[tBid^{MOM}][Bcl2] + k_{f4}[Bax^{MOM} \cdot Bcl2] - k_4[Bax^{MOM}][Bcl2] + k_{f18}[Puma^{MOM} \cdot Bcl2] - k_{18}[Puma^{MOM}][Bcl2]$$

$$(6)\quad \frac{d[Bax^{MOM} \cdot Bcl2]}{dt} = k_4[Bax^{MOM}][Bcl2] - k_{f4}[Bax^{MOM} \cdot Bcl2] - k_{f11}[Bax^{MOM} \cdot Bcl2]$$

$$(7)\quad \frac{d[tBid^{MOM} \cdot Bcl2]}{dt} = k_2[tBid^{MOM}][Bcl2] - k_{f2}[tBid^{MOM} \cdot Bcl2] - k_{f10}[tBid^{MOM} \cdot Bcl2] + k_{f26}[A1 \cdot tBid] - k_{26}[A1][tBid]$$

$$(8)\quad \frac{d[Bax^{MOM} \cdot Bax^{MOM}]}{dt} = k_{12}[Bax^{MOM}]^2 - k_{f12}[Bax^{MOM} \cdot Bax^{MOM}] + 2k_{f14}[(Bax^{MOM})^2 \cdot (Bax^{MOM})^2] - 2k_{14}[Bax^{MOM} \cdot Bax^{MOM}]^2 - k_{f13}[Bax^{MOM} \cdot Bax^{MOM}]$$

$$(9)\quad \frac{d[(Bax^{MOM})^2 \cdot (Bax^{MOM})^2]}{dt} = k_{14}[Bax^{MOM} \cdot Bax^{MOM}]^2 - k_{f14}[(Bax^{MOM})^2 \cdot (Bax^{MOM})^2] - k_{f15}[(Bax^{MOM})^2 \cdot (Bax^{MOM})^2]$$

$$(10)\quad \frac{d[Puma]}{dt} = k_{17} - k_{f17}[Puma] - k_{Pumaon}[Puma] + k_{Pumaoff}[Puma^{MOM}] + k_{f25}[A1 \cdot Puma] - k_{25}[A1][Puma]$$

$$(11)\quad \frac{d[Puma^{MOM} \cdot Bcl2]}{dt} = k_{18}[Puma^{MOM}][Bcl2] - k_{f18}[Puma^{MOM} \cdot Bcl2] - k_{f19}[Puma^{MOM} \cdot Bcl2]$$

$$(12)\quad \frac{d[tBid^{MOM}]}{dt} = Beta\big(k_{tBidMOM}[tBid] - k_{tBidoff}[tBid^{MOM}]\big) - k_{f8}[tBid^{MOM}] - k_2[tBid^{MOM}][Bcl2] + k_{f2}[tBid^{MOM} \cdot Bcl2]$$

$$(13)\quad \frac{d[Bax^{MOM}]}{dt} = Beta\big(k_{Baxon}[Bax^*] - k_{Baxoff}[Bax^{MOM}]\big) - k_{f9}[Bax^{MOM}] + k_{f4}[Bax^{MOM} \cdot Bcl2] - k_4[Bax^{MOM}][Bcl2] + 2k_{f12}[Bax^{MOM} \cdot Bax^{MOM}] - 2k_{12}[Bax^{MOM}]^2$$

$$(14)\quad \frac{d[Puma^{MOM}]}{dt} = Beta(\ k_{Pumaon}[Puma] - k_{Pumaoff}[Puma^{MOM}]) - k_{18}[Puma^{MOM}][Bcl2] + k_{f18}[Puma^{MOM} \cdot Bcl2] - kd_{Puma}[Puma^{MOM}]$$

$$(15)\quad \frac{d[A1]}{dt} = k_{A1} - k_{dA1}[A1] + k_{f24}[A1 \cdot Bax^*] - k_{24}[A1][Bax^*] + k_{f25}[A1 \cdot Puma] - k_{25}[A1][Puma] + k_{f26}[A1 \cdot tBid] - k_{26}[A1][tBid] + k_{f27}[A1 \cdot Noxa] - k_{27}[A1][Noxa]$$

$$(16)\quad \frac{d[Noxa]}{dt} = k_{Noxa} - k_{dNoxa}[Noxa] + k_{f27}[A1 \cdot Noxa] - k_{27}[A1][Noxa]$$

$$(17)\quad \frac{d[A1 \cdot Bax^*]}{dt} = k_{24}[A1][Bax^*] - k_{f24}[A1 \cdot Bax^*] - k_{dA1Bax*}[A1 \cdot Bax^*]$$

$$(18)\quad \frac{d[A1 \cdot Puma]}{dt} = k_{25}[A1][Puma] - k_{f25}[A1 \cdot Puma] - k_{dA1Puma}[A1 \cdot Puma]$$

$$(19)\quad \frac{d[A1 \cdot tBid]}{dt} = k_{26}[A1][tBid] - k_{f26}[A1 \cdot tBid] - k_{dA1tBid}[A1 \cdot tBid]$$

$$(20)\quad \frac{d[A1 \cdot Noxa]}{dt} = k_{27}[A1][Noxa] - k_{f27}[A1 \cdot Noxa] - k_{dA1Noxa}[A1 \cdot Noxa]$$

1.2Parameters

Parameter values are chosen with the same scale, which are estimated from available experiments and/or previous models. The concentration unit is nM and the time unit is s.

Table A

Parameter	Value/Unit	Description	Corresponding interaction	Source
kdBax	1e-4 s^{-1}	Degradation rate of activated Bax	bax*_deg	(2)

kdtBid	1e-4 s^{-1}	Degradation rate of tBid	tbid_deg	Same as kdBax
k3	1e-5 $nM^{-1}s^{-1}$	Activation rate of Bax by membrane binding tBid	tbidMOM_act_bax	(1)
k16	2.2e-5 $nM^{-1}s^{-1}$	Activation rate of Bax by membrane binding Bax dimer	baxMOM^2_act_bax	Estimated from k3
k2	4e-4 $nM^{-1}s^{-1}$	Association rate of membrane binding tBid and Bcl2	tbidMOM * bcl2	(3)
kf2	2e-3 s^{-1}	Dissociation rate of membrane binding tBid and Bcl2	tbidMOM _bcl2	(3)
k4	2e-4 $nM^{-1}s^{-1}$	Association rate of membrane binding Bax and Bcl2	baxMOM * bcl2	(3)
kf4	3e-3 s^{-1}	Dissociation rate of membrane binding Bax and Bcl2	baxMOM_bcl2	(3)
k12	2e-4 $nM^{-1}s^{-1}$	Association rate of membrane binding Bax	baxMOM*baxMOM	Estimated from k4
kf12	3e-3 s^{-1}	Dissociation rate of membrane binding Bax	baxMOM_baxMOM	Estimated from kf4
k14	2e-4 $nM^{-1}s^{-1}$	Association rate of membrane binding Bax dimer	baxMOM^2 * baxMOM^2	Estimated from k4
kf14	3e-3 s^{-1}	Dissociation rate of membrane binding Bax dimer	baxMOM^2_baxMOM^2	Estimated from kf4
k18	5e-4 $nM^{-1}s^{-1}$	Association rate of membrane binding Puma and Bcl2	pumaMOM * bcl2	(3)
kf18	2e-3 s^{-1}	Dissociation rate of membrane binding Puma and Bcl2	pumaMOM_bcl2	(3)
k5	2e-3 nM/s	Production rate of Bid	bid_pro	Estimated from (2)
kf5	1e-4 s^{-1}	Degradation rate of Bid	bid_deg	Same as kdBax
k6	2e-2 nM/s	Production rate of Bax	bax_pro	Estimated from (2)
kf6	1e-4 s^{-1}	Degradation rate of Bax	bax_deg	Same as kdBax
k7	8e-3 nM/s	Production rate of Bcl2	bcl2_pro	Estimated from (2)

kf7	1e-4 s^{-1}	Degradation rate of Bcl2	bcl2_deg	Same as kdBax
k17	1e-3 nM/s	Production rate of Puma	puma_pro	Estimated
kf17	1e-4 s^{-1}	Degradation rate of Puma	puma_deg	Same as kdBax
kf8	1e-4 s^{-1}	Degradation rate of membrane binding tBid	tbidMOM_deg	Same as kdBax
kf9	1e-4 s^{-1}	Degradation rate of membrane binding Bax	baxMOM_deg	Same as kdBax
kf10	1e-4 s^{-1}	Degradation rate of Bcl2·tBidMOM complex	bcl2·tbidMOM_deg	Same as kdBax
kf11	1e-4 s^{-1}	Degradation rate of Bcl2·BaxMOM complex	bcl2·baxMOM_deg	Same as kdBax
kf13	1e-4 s^{-1}	Degradation rate of Bax dimer	baxMOM·baxMOM_deg	Same as kdBax
kf15	1e-4 s^{-1}	Degradation rate of Bax oligomer	baxMOM^2·baxMOM^2_deg	Same as kdBax
kf19	1e-4 s^{-1}	Degradation rate of Bcl2·tBidMOM complex	bcl2·pumaMOM_deg	Same as kdBax
kBidon	2e-3 s^{-1}	Membrane translocation rate of tBid	tbid_onMOM	Estimated from cell size
kBidoff	2e-4 s^{-1}	Membrane separation rate of tBid	tbid_offMOM	Estimated from cell size
kBaxon	2e-3 s^{-1}	Membrane translocation rate of active Bax	bax*_onMOM	Same as kBidon
kBaxoff	2e-3 s^{-1}	Membrane separation rate of active Bax	bax*_offMOM	Same as kBidoff
kPumaon	2e-2 s^{-1}	Membrane translocation rate of Puma	puma_onMOM	Same as kBidon
kPumaoff	2e-4 s^{-1}	Membrane separation rate of Puma	puma_offMOM	Same as kBidoff
kdPuma	1e-4 s^{-1}	Degradation rate of membrane binding Puma	pumaMOM_deg	Same as kdBax
k24	4e-4 $nM^{-1}s^{-1}$	Association rate of A1 and active Bax	A1 * bax*	(3)
kf24	4e-3 s^{-1}	Dissociation rate of A1 and active Bax	A1_bax*	(3)
k25	5e-4 $nM^{-1}s^{-1}$	Association rate of A1 and Puma	A1 * puma	(3)

kf25	2e-3 s^{-1}	Dissociation rate of A1 and Puma	A1 - puma	(3)
k26	2e-4 $nM^{-1}s^{-1}$	Association rate of A1 and tBid	A1 * tbid	(3)
kf26	1e-3 s^{-1}	Dissociation rate of A1 and tBid	A1 - tbid	(3)
k27	1.5e-6 $nM^{-1}s^{-1}$	Association rate of A1 and Noxa	A1 * noxa	(3)
kf27	3e-3 s^{-1}	Dissociation rate of A1 and Noxa	A1 - noxa	(3)
kA1	3e-3 nM/s	Production rate of A1	A1_pro	Estimated from (2)
kdA1	1e-4 s^{-1}	Degradation rate of A1	A1_deg	Same as kdBax
kdA1Bax	1e-4 s^{-1}	Degradation rate of A1·Bax complex	A1·bax_deg	Same as kdBax
kdA1Puma	1e-4 s^{-1}	Degradation rate of A1·Puma complex	A1·puma_deg	Same as kdBax
kdA1tBid	1e-4 s^{-1}	Degradation rate of A1·tBid complex	A1·tbid_deg	Same as kdBax
kdA1Noxa	1e-4 s^{-1}	Degradation rate of A1·Noxa complex	A1·noxa_deg	Same as kdBax
kNoxa	1e-3	Production rate of Noxa	Noxa_pro	Estimated from (2)
kdNoxa	1e-4 s^{-1}	Degradation rate of Noxa	Noxa_deg	Same as kdBax
Beta	1	Constant of cell volume normalization	—	—
k1	1e-6 $nm^{-1}s^{-1}$	Activation rate of Bid by caspase8	—	Estimated from k3

1.3 Local sensitivity analysis in ODEs

We extended the parameter perturbation degree from the original 2% to 1, 5 and 10%. It shows that the sensitivity rank of each parameter and the sensitivity patterns are approximately the same among the spectrums.

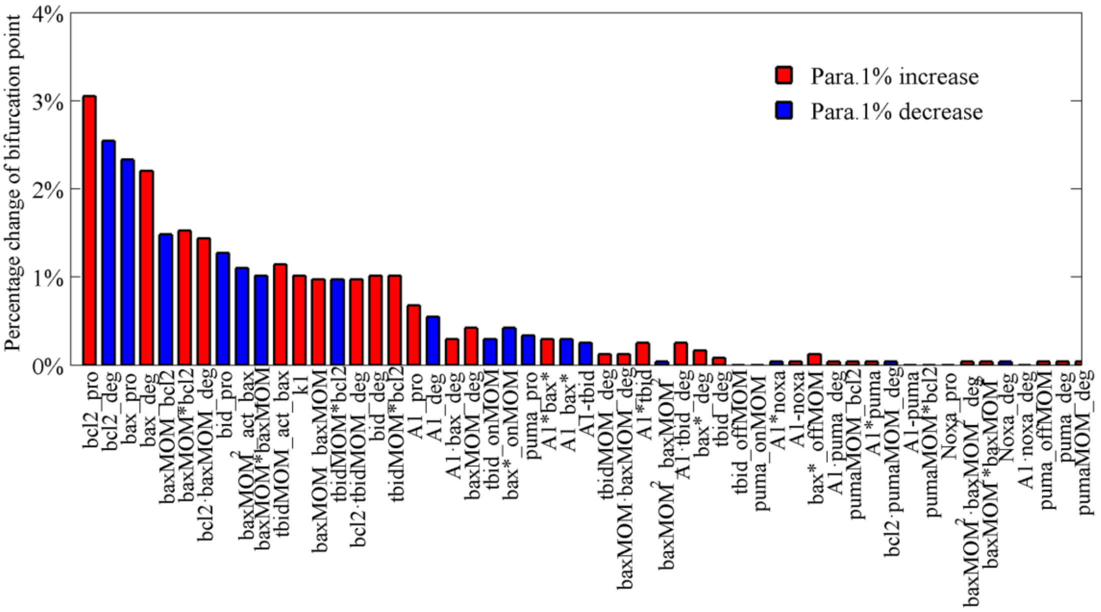

Fig A. Parameter sensitivity spectrum in deterministic simulation.

1% decrease or increase of each parameter induced the percentage change of the bifurcation point SN with local sensitivity analysis method. Horizontal coordinates represent parameters of the model, vertical coordinates stand for the percent changes of bifurcation-point location in response to the corresponding changes of parameters.

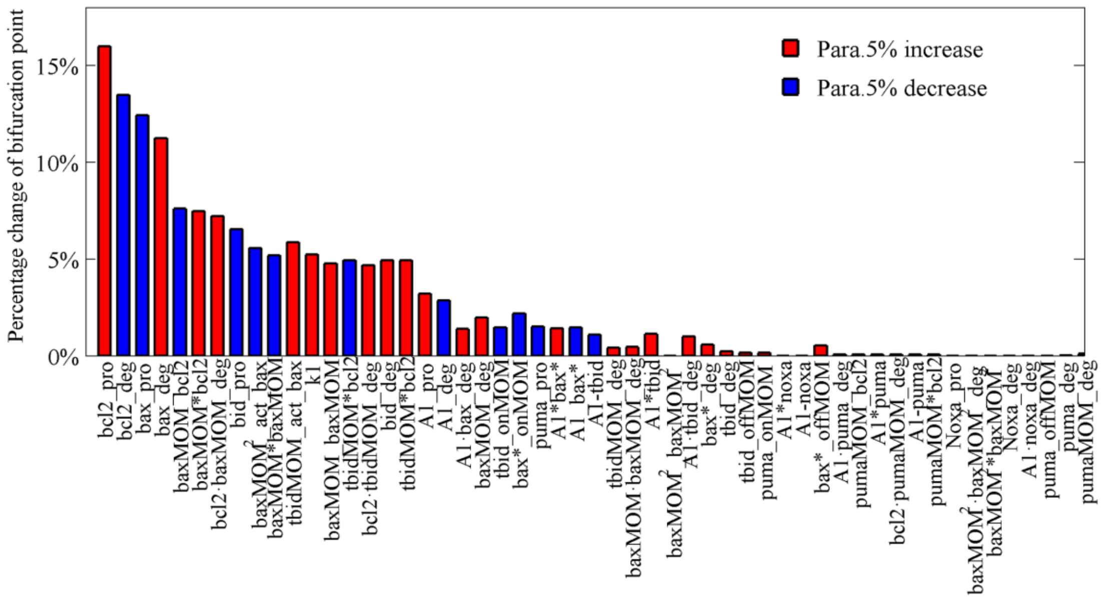

Fig B. Parameter sensitivity spectrum in deterministic simulation.

5% decrease or increase of each parameter induced the percentage change of the bifurcation point SN with local sensitivity analysis method. Horizontal coordinates represent parameters

of the model, vertical coordinates stand for the percent changes of bifurcation-point location in response to the corresponding changes of parameters.

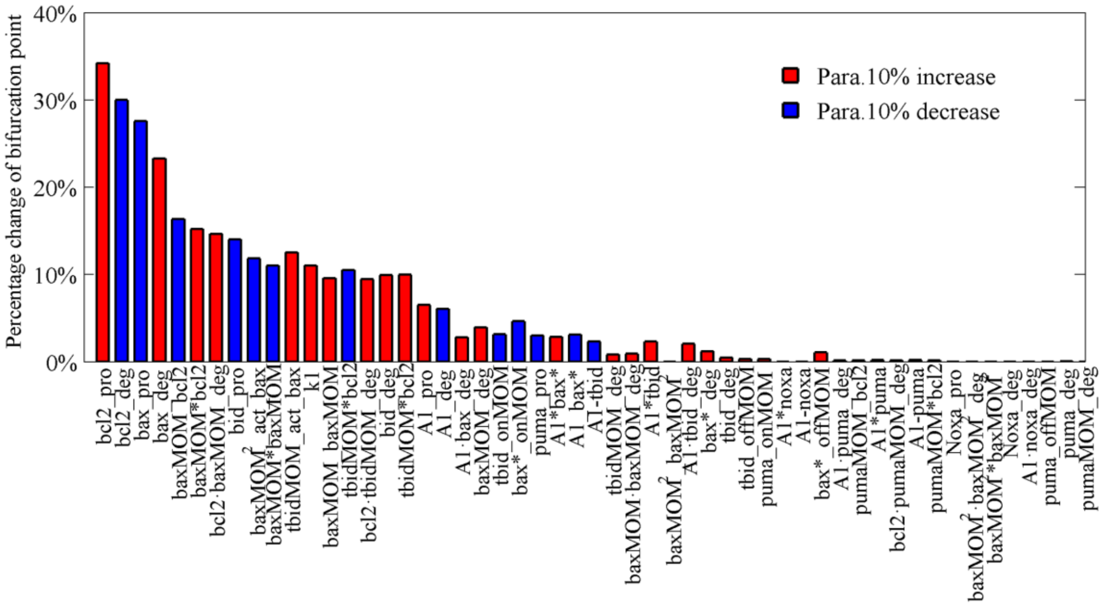

Fig C. Parameter sensitivity spectrum in deterministic simulation.

10% decrease or increase of each parameter induced the percentage change of the bifurcation point SN with local sensitivity analysis method. Horizontal coordinates represent parameters of the model, vertical coordinates stand for the percent changes of bifurcation-point location in response to the corresponding changes of parameters.

1.4 Gillespie Stochastic Simulation Algorithm

There are 20 molecular species (X1(t), X2(t) …X20(t)) with 53 elementary reaction channels in our model. Number of molecular Xi is function of time t. $\frac{dX_i(t)}{dt} = V\frac{dx_i(t)}{dt}$; x_i is protein concentration in ODEs; V is the reaction volume of the system. In our simulation, first passage-time (FPT) is identified as the time when the Bax4 MOM number first reaches 80% of mean value of high state. Other thresholds like 50% and 90% of mean value of high state that characterize the change of low to high steady states are also used.

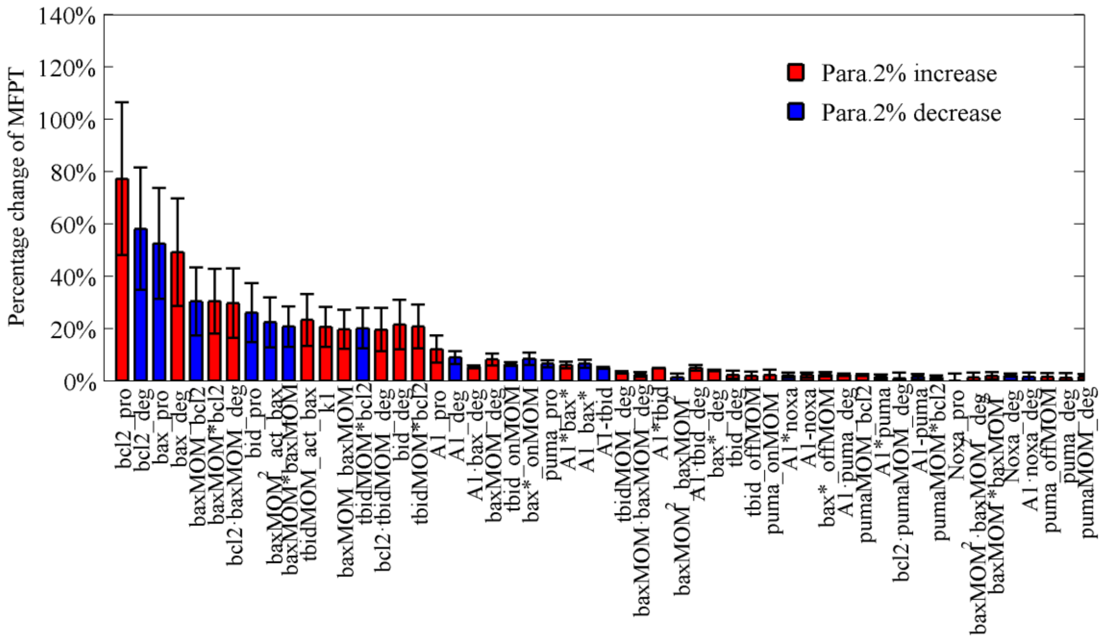

Fig D. Parameter sensitivity analysis in SSA with a threshold of 50% of mean value of high state. Red histograms illustrate changes of MFPT in response to 2% increase of parameters. Blue histograms represent changes of MFPT in response to 2% decrease of parameters. Dark bars are coefficients of variance. Increasing and decreasing are chosen so that MFPT gets longer. Details of the correspondence between parameters and represented interactions were shown in Table. S1. Asterisk, association; deg, degradation rate; onMOM/offMOM, membrane translocation and membrane separation; pro, production rate; minus, dissociation.

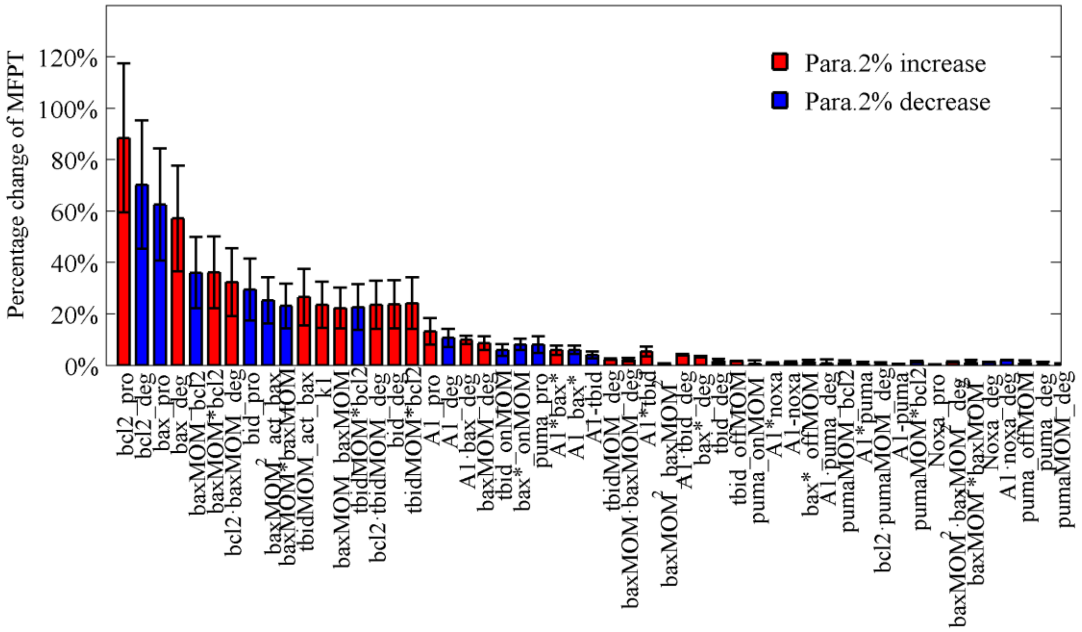

Fig E. Parameter sensitivity analysis in SSA with a threshold of 90% of mean value of high state. Red histograms illustrate changes of MFPT in response to 2% increase of parameters. Blue histograms represent changes of MFPT in response to 2% decrease of parameters. Dark bars are coefficients of variance. Increasing and decreasing are chosen so that MFPT gets longer. Details of the correspondence between parameters and represented interactions were shown in Table. S1. Asterisk, association; deg, degradation rate; onMOM/offMOM, membrane translocation and membrane separation; pro, production rate; minus, dissociation.

We choose V=5, V= 50, V=100 in our stochastic simulations. The initial conditions are set to low steady state which is calculated from ODEs, indicating that the cell starts from a non-apoptosis state. Production rates of proteins whose unit is nM/s in ODEs need multiply by V in SSA. Degradation rates and dissociation rates of proteins whose unit is s-1 don't change. Association rates need divide by V. We have focused on changes of Bax oligomers.

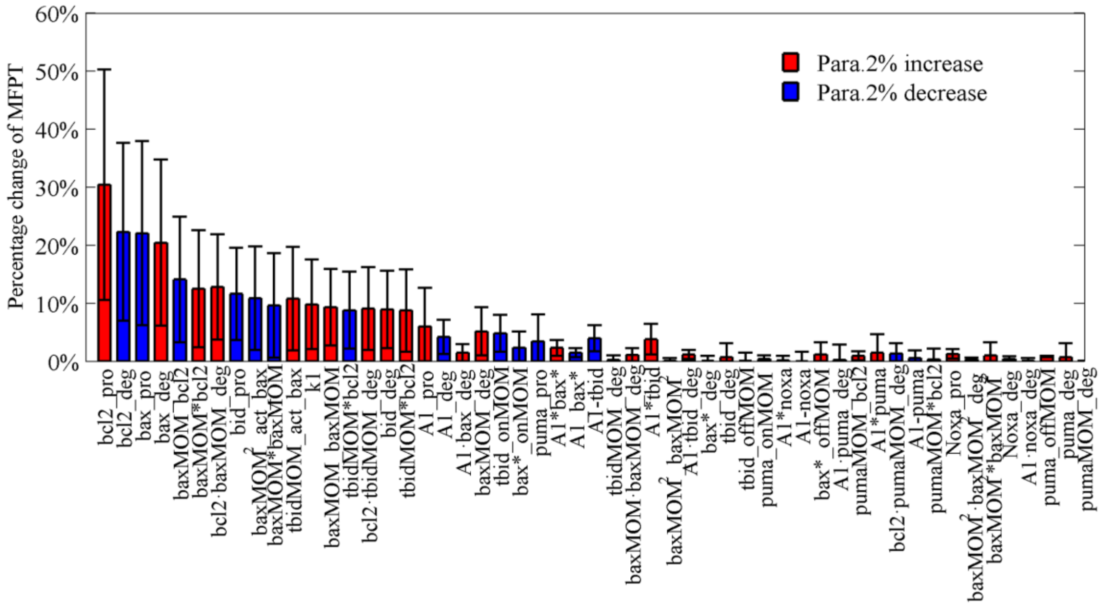

Fig F. Parameter sensitivity analysis in Gillespie stochastic simulations with V=5. Red histograms illustrate changes of MFPT in response to 2% increase of parameters. Blue histograms represent changes of MFPT in response to 2% decrease of parameters. Dark bars are coefficients of variance. Increasing and decreasing are chosen so that MFPT gets longer. Details of the correspondence between parameters and represented interactions were shown in Table. S1. Asterisk, association; deg, degradation rate; onMOM/offMOM, membrane translocation and membrane separation; pro, production rate; minus, dissociation.

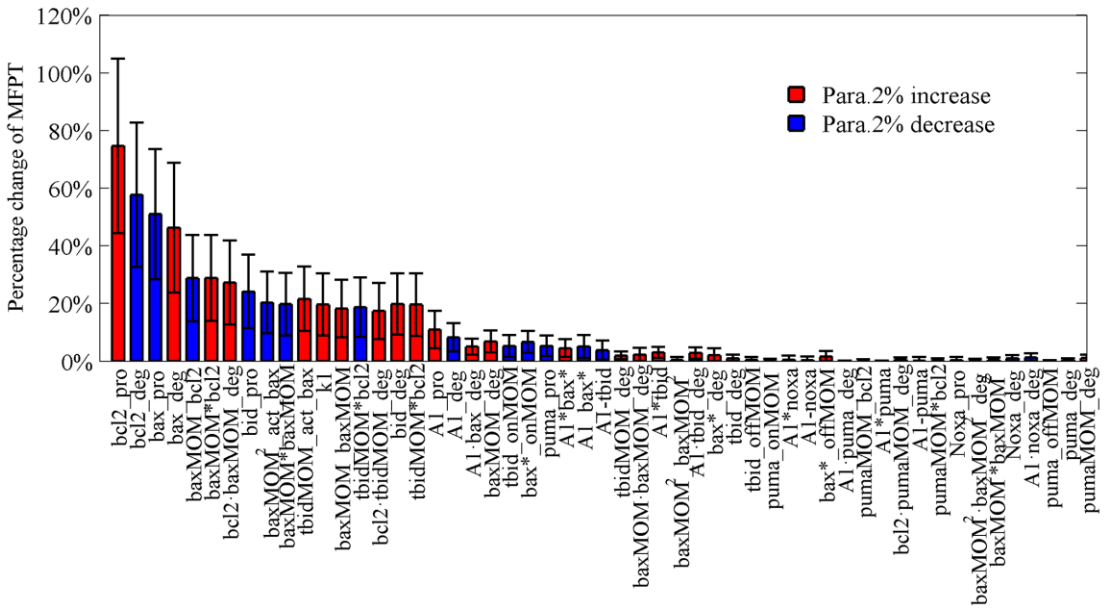

Fig G. Parameter sensitivity analysis in Gillespie stochastic simulations with V=50.

Red histograms illustrate changes of MFPT in response to 2% increase of parameters. Blue

histograms represent changes of MFPT in response to 2% decrease of parameters. Dark bars are coefficients of variance. Increasing and decreasing are chosen so that MFPT gets longer. Asterisk, association; deg, degradation rate; onMOM/offMOM, membrane translocation and membrane separation; pro, production rate; minus, dissociation.

1.5 The quasi-potential energy landscape

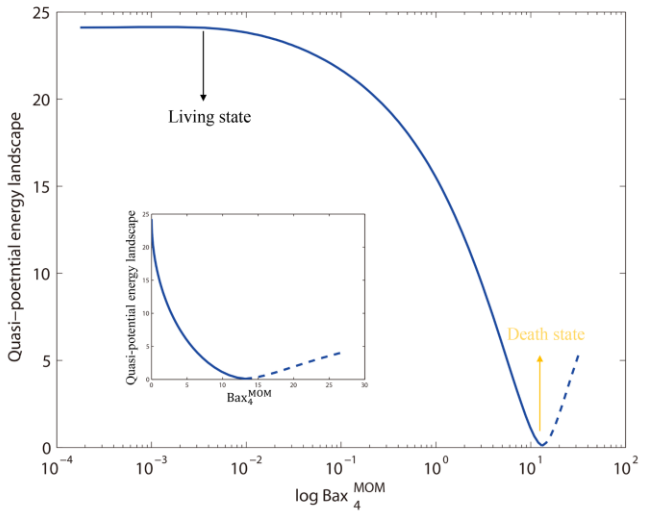

Fig H. The quasi-potential energy landscape in terms of log $Bax_4{}^{MOM}$ when Cas8=22nM. The small figure inserted shows the quasi-potential energy landscape in terms of $Bax_4{}^{MOM}$. The two figures describe the same thing but with different abscissa variables ($Bax_4{}^{MOM}$ and log $Bax_4{}^{MOM}$). The potential well of living state becomes very shallow when Cas8=22nM which is near the bifurcation point. Though it's hard to see the local minimum of living state in the picture, the local minimum does exist when we amplify it.

1.6 Spearman rank correlation of parameter sensitivity with different methods.

	Gillespie SSA (C8=23nM)	ODE	gMAM (C8=22nM)	gMAM (C8=4.9nM)
Gillespie SSA (C8=23)	1	0.99	0.973	0.827
ODE		1	0.981	0.852
gMAM (C8=22）			1	0.826
gMAM (C8=4.9）				1

Significance levels are all less than 0.001.

Table B. Spearman rank correlation of parameter sensitivity with different methods.
C8 stands for Caspase8. The location of the saddle-node (SN) bifurcation point is Caspase8=23.59nM. So c8=23 and c8=22 is close to the bifurcation point and c8=4.9 is far from the bifurcation point.

Comparing Spearman rank correlations of parameter sensitivity as shown in Table B, we can see that parameter sensitivity spectrum according to bifurcation point position is more similar with spectrum got by energy barrier that near bifurcation point.

1.7 A Short Introduction to Quasi-potential Landscape for Markov Jump Process

The landscape provides a vivid pictorial description as well as an insightful qualitative tool to understand highly complex stochastic multi-stable system. There are many candidates of landscape functions that can provide quantitative understanding about stochastic systems such as the relative stability of different attracters, transition rates between metastable states etc. The connections between different landscapes and their basic properties can be found in Ref. [4].

Quasi-potential comes from large deviation theory. When the system size is large enough, that is, the noise is small enough, the large deviation theory tells that for any path $\varphi \in C[0,T]$,

$$\mathrm{Prob}\left(\sup_{0\le t\le T}\left|\frac{X_t}{V}-\varphi\right|\le\delta\right)\approx\exp(-VS_T[\varphi]).$$

That is to say, the probability that the trajectory of stochastic process x_t lies in a neighborhood of a given path can be approximated by a functional $S_T[\varphi]$. The functional is defined as

$$S_T[\varphi]=\int_0^T L(\varphi,\dot{\varphi})\,dt.$$

There is no close form of the Lagrangian $L(\varphi,\dot{\varphi})$ for chemical jump processes. But its duality can be written down explicitly.

$$H(x,p)=\sum_j a_j(x)(e^{p\cdot v_j}-1),$$

$$L(x,y)=\sup_p\{p\cdot y-H(x,p)\}.$$

Assume x_0 is a stable state of deterministic process $\dot{x} = \sum_j a_j(x)\nu_j$, the local quasi-potential with respect to x_0 is defined as

$$S(x; x_0) = \inf_{T>0} \inf_{\varphi(0)=x_0, \varphi(T)=x} S_T[\varphi]$$

The intuitive explanation of local quasi-potential is that the least cost of action of moving the system from x_0 to x. So intuitively, local quasi-potential reflects the difficulty of transition within a single attractor. The larger the potential is, the more difficult the system transits from x_0 to x. For x that is out of the attractor of x_0, local quasi-potential measures the stability of attractor with respect to x_0. The larger the potential is, the more difficult the system escapes from the attractor of x_0. Indeed, these properties can be proved rigorously. One may refer to Ref. [5] for the proof.

For x_0 and x fixed, recall

$$\mathrm{Prob}\left(\sup_{0\le t\le T}\left|\frac{X_t}{V} - \varphi\right| \le \delta\right) \approx \exp(-VS_T[\varphi])$$

We know that when system size is large enough, the minimum of $S_T[\varphi]$ dominates the probability that X_t/V transits from x_0 to x. The minimizer of $S_T[\varphi]$ dominates the transition path from x_0 to x. So the procedure of finding local quasi-potential also characterizes the essential mechanics of transition.

The global quasi-potential can be constructed from local ones by a pruning and sticking procedure. In Ref. [4], there is a simple but clear example of this procedure. The sticking procedure determines the relative stability of different stable states, so that one can figure out the relative stability and the difficulty of transition between any pair of points in the whole space.

1.8 Variance-based Sobol sensitivity analysis

Results of system biology largely depend on initial conditions and parameters. Unfortunately, Parameters used in the model are usually difficult or even impossible to get from experimental data [6]. Sensitivity analysis as a powerful tool in system biology is often applied to determine how robust the biological response to parameter changes. Sensitivity analysis can be classified into local sensitivity analysis (LSA) and global sensitivity analysis (GSL). LSA methods are commonly utilized to analyze the behavior

of the model output in the vicinity of a chosen point. They are usually efficient in computer time but may be inadequate for nonlinear models. GSA methods allow one to explore the full-phase space of input parameters and to take the nonlinearity of the model into account [7].

Variance-based Sobol sensitivity analysis is a common method of GSL. Variance-based Sobol sensitivity analysis makes no assumptions about the relations between models' inputs and outputs. Outputs function can be decomposed into summands of variances in combinations of input parameters.

$\mathrm{Y} = f_0 + \sum_{i=1}^{d} f_i(X_i) + \sum_{i<j}^{d} f_{ij}\left(X_i, X_j\right) + \cdots + f_{1\cdots k}(X_1, X_2, \cdots X_d)$, where f_0 is a constant and f_i is a function of x_i, f_{ij} a function of x_i and x_j.

$$f_0 = \mathrm{E(Y)}$$

$$f_i(X_i) = E(Y|X_i) - f_0$$

$$f_{ij}\left(X_i, X_j\right) = \mathrm{E}\left(\mathrm{Y}\middle|X_i, X_j\right) - f_0 - f_i - f_j$$

The total variance is defined as :

$\mathrm{Var(Y)} = \sum_{i=1}^{d} V_i + \sum_{i<j}^{d} V_{ij} + \cdots + V_{12\cdots d}$

where

$$V_i = Var_{X_i}(E_{X_{\sim i}}(Y|X_i),$$

$$V_{ij} = Var_{X_{ij}}(E_{X_{\sim ij}}\left(Y\middle|X_i, X_j\right) - V_i - V_j$$

First-order sensitivity index is stated as follows,

$$S_i = \frac{V_i}{Var(Y)}$$

Total-effect index is given as: $S_{Ti} = \frac{E_{X_{\sim i}}(Var_{X_i}(Y|X_{\sim i})}{Var(Y)} = 1 - \frac{Var_{X_{\sim i}}(E_{X_i}(Y|X_{\sim i})}{Var(Y)}$

1.9 Equivalence of the parameter sensitivity by bifurcation-point position analysis and barrier height analysis for Schlögl model

We will focus on an autocatalytic, tri-molecular reaction scheme, first proposed by Schlögl (Schlögl, F. Z. Physik. 1972),

$$\mathrm{A} + 2\mathrm{X} \underset{k_2}{\overset{k_1}{\rightleftharpoons}} 3\mathrm{X},$$

$$\mathrm{B} \underset{k_4}{\overset{k_3}{\rightleftharpoons}} \mathrm{X}.$$

Let a and b denote the concentrations of the chemicals A and B, respectively, which are set as constants in the following studies. Let x be the concentration of the chemical X. The deterministic model based on the law of mass action is a first-order, nonlinear ODE:

$$\frac{dx}{dt} = k_1 a x^2 - k_2 x^3 - k_4 x + k_3 b. \tag{1}$$

For ease of notation, we still denote $k_1 a$ by k_1, and $k_3 b$ by k_3 in theoretical analysis. Define $f(\mathrm{x}) = k_1 x^2 - k_2 x^3 - k_4 x + k_3$. We will show that the equivalence indeed holds for the parameter sensitivity obtained by deterministic bifurcation-point position analysis and barrier height analysis if k_3 is chosen as the target parameter.

(1) Parameter sensitivity analysis according to the bifurcation-point position

At the bifurcation point x_0 we have $f(x_0) = 0$ and $\frac{df}{dx}(x_0) = 0$. Then we get:

$$k_1 {x_0}^2 - k_2 {x_0}^3 - k_4 x_0 + k_3 = 0 \tag{2}$$

and

$$2k_1 x_0 - 3k_2 {x_0}^2 - k_4 = 0. \tag{3}$$

We take k_3 as the control parameter to achieve the bi-stability (like Caspase8 in our mitochondrial apoptosis model). By solving equations (2) and (3), we obtain the location of the bifurcation points and the corresponding k_3:

$$x_0 = \frac{k_1 \pm \sqrt{{k_1}^2 - 3k_2 k_4}}{3k_2}, \tag{4}$$

$$k_3 = k_2 {x_0}^3 - k_1 {x_0}^2 + k_4 x_0, \tag{5}$$

where $\pm$ means that there are two bifurcation points in this system.

The partial derivatives of k_3 with respect to the remaining three parameters are:

$$\frac{\partial k_3}{\partial k_1} = -{x_0}^2 + (3k_2 {x_0}^2 - 2k_1 x_0 + k_4)\frac{\partial x_0}{\partial k_1}$$

$$\frac{\partial k_3}{\partial k_2} = {x_0}^3 + (3k_2 {x_0}^2 - 2k_1 x_0 + k_4)\frac{\partial x_0}{\partial k_2}$$

$$\frac{\partial k_3}{\partial k_4} = x_0 + (3k_2 {x_0}^2 - 2k_1 x_0 + k_4)\frac{\partial x_0}{\partial k_4}$$

Based on Eqn. (3), the above three equations can be simplified to:

$$\frac{\partial k_3}{\partial k_1} = -{x_0}^2 \tag{6}$$

$$\frac{\partial k_3}{\partial k_2} = {x_0}^3 \tag{7}$$

$$\frac{\partial k_3}{\partial k_4} = x_0. \tag{8}$$

The sensitivity of the parameter k_3 corresponding to the bifurcation point in response to parameter changes are defined as:

$$\gamma_{k_1} = \frac{k_3(k_1 + \Delta k_1) - k_3}{k_3} \approx \frac{\frac{\partial k_3}{\partial k_1}\Delta k_1}{k_3}$$

$$\approx -{x_0}^2 \Delta k_1 / k_3$$

$$\gamma_{k_2} \approx {x_0}^3 \Delta k_2 / k_3$$

$$\gamma_{k_4} \approx x_0 \Delta k_4 / k_3$$

where $k_3(\cdot)$ stands for the position of k_3 at the bifurcation point, and Δk_i is the magnitude of i-th parameter perturbation.

When same amount of relative parameter perturbations are applied, i.e. the same $\Delta k_i / k_i$, we can get the following simplified form by ignoring a common factor, which does not affect the ranking of sensitivity:

$$\gamma_{k_1} \sim -{x_0}^2 k_1 \quad (9)$$

$$\gamma_{k_2} \sim {x_0}^3 k_2 \quad (10)$$

$$\gamma_{k_4} \sim x_0 k_4 \quad (11)$$

From Eqn.s (9)-(11) we can see that the sensitivity spectrums are different for different parameters. And we may get different parameter sensitivity spectrums corresponding to different bifurcation points (There are actually two bifurcation points x_0 for this bi-stable system).

(2) Parameter sensitivity analysis according to the barrier height

Now we add the white noise to the dynamics (1) to construct a stochastic Schlögl model

$$\frac{dx}{dt} = f(x) + \sqrt{2\varepsilon}\dot{W}_t$$

There are three stationary points as shown in previous subsection. Two of them are stable fixed points x_{st1}, x_{st2} and the other is an unstable fixed point x_{unst} . These three points satisfy $x_{st1} < x_{unst} < x_{st2}$.

The quasi-potential of stochastic Schlögl model is:

$$V(x) = -\int f(x)dx$$

$$= \frac{k_2 x^4}{4} - \frac{k_1 x^3}{3} + \frac{k_4 x^2}{2} - k_3 x$$

Denote the values of the potential at the stable and unstable fixed points by $V(x_{st1})$, $V(x_{st2})$ and $V(x_{unst})$. The barrier height that needs to climb from stable fixed point x_{st1} to x_{st2} is:

$\Delta V_1 = V(x_{unst}) - V(x_{st1})$

(12)

Correspondingly, the barrier height from x_{st2} to x_{st1} is:

$\Delta V_2 = V(x_{unst}) - V(x_{st2})$.

(13)

The partial derivatives of ΔV_i to the three parameters are:

$$\frac{\partial \Delta V_i}{\partial k_1} = -\frac{x_{unst}^3 - x_{sti}^3}{3} \quad (14)$$

$$\frac{\partial \Delta V_i}{\partial k_2} = \frac{x_{unst}^4 - x_{sti}^4}{4} \quad (15)$$

$$\frac{\partial \Delta V_i}{\partial k_4} = \frac{x_{unst}^2 - x_{sti}^2}{2} \quad (16)$$

The parameter sensitivity of ΔV_i with respect to parameter k_j can be defined as: $Vi_{k_j} = \frac{\Delta V_i(k_j+\Delta k_j)-\Delta V_i}{\Delta V_i}$. Following similar derivations as previous subsection, we get:

$$V_{i,k_1} \approx -\frac{x_{unst}^3 - x_{sti}^3}{3} k_1 \quad (17)$$

$$V_{i,k_2} \approx \frac{x_{unst}^4 - x_{sti}^4}{4} k_2 \quad (18)$$

$$V_{i,k_4} \approx \frac{x_{unst}^2 - x_{sti}^2}{2} k_4 \quad (19)$$

Since the analytic solutions of the fixed points are complex, we only consider the case when the system is close to the tipping point. With out loss of generality, take *i=1*. By the mean value theorem, we have

$$\frac{\partial \Delta V_i}{\partial k_1} = -\xi_1^2 (x_{unst} - x_{st1})$$

$$\frac{\partial \Delta V_i}{\partial k_2} = \xi_2^3 (x_{unst} - x_{st1})$$

$$\frac{\partial \Delta V_i}{\partial k_4} = \xi_4 (x_{unst} - x_{st1})$$

where $x_{st1} < \xi_j < x_{unst}, j = 1,2,4$. When considering the ranking of parameter sensitivity, we can ignore the common constant $x_{unst} - x_{st1}$. Since the parameter is close to the bifurcation point, we have $\xi_1, \xi_2, \xi_4 \approx x_0$ and thus

$$V_{1,k_1} \sim -x_0^2 k_1 \quad (20)$$

$$V_{1,k_2} \sim x_0^3 k_2 \quad (21)$$

$$V_{1,k_4} \sim x_0 k_4. \quad (22)$$

It is the same as Eqn.s (9)-(11). This shows that when the parameter is sufficiently close to the tipping point, the ranking of sensitivity by barrier height analysis is the same as that obtained by the bifurcation-point position analysis.

(3) Numerical simulations

As an example, we take $k_1 = 2.4, k_2 = 0.54, k_4 = 2.95$, $k_3$as the input and the value of *x* as the output to plot the bifurcation diagram (as shown in Fig I).

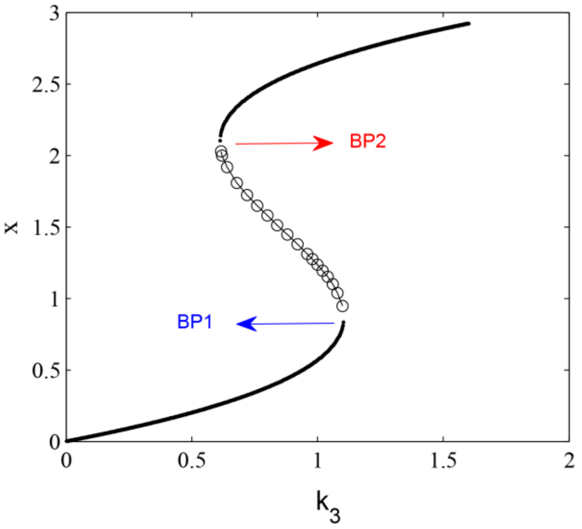

Fig I. k_3 **is taken as the control parameter, and** ***x*** **as the output.** BP indicates bifurcation point. There are two bifurcation points in the model. The coordinates of the first bifurcation point (BP1) are (1.104, 0.8312) and the coordinates of the second bifurcation point (BP2) are (0.612, 2.103).

Then we conduct the parameter sensitivity analysis according to the change of BP1 and BP2 (as shown in Fig J).

(a)

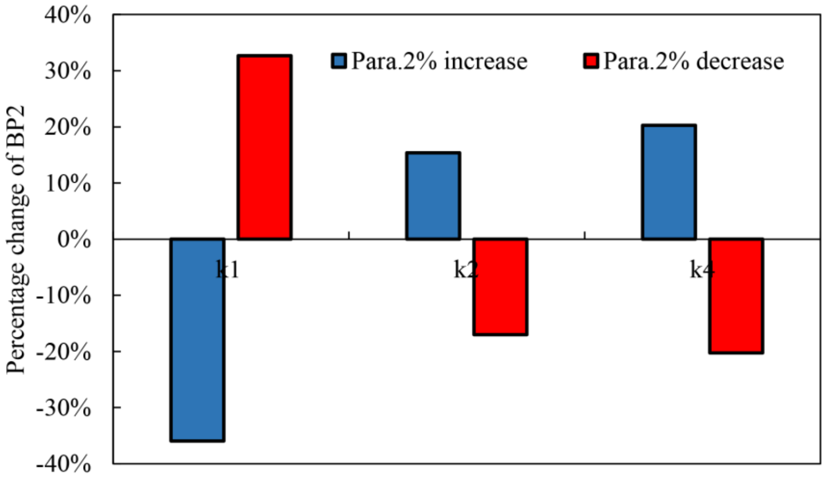

(b)

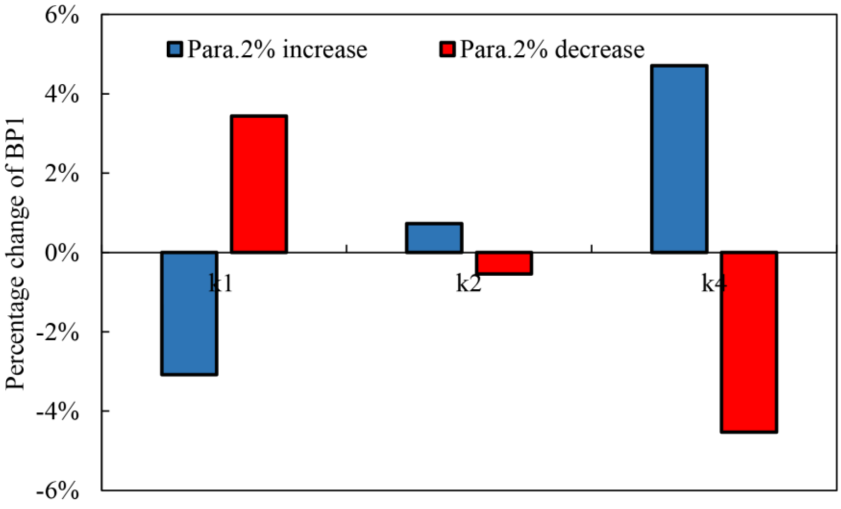

Fig J. Parameter sensitivity spectrum in deterministic simulation.

(a) 2% decrease and increase in each parameter induced a percentage change of the BP1. The horizontal axis represents the parameters of the model, and the vertical axis represents percent changes of bifurcation-point location in response to the corresponding changes in the

parameters. (b) A 2% decrease and increase in each parameter induced a percentage change of BP2.

From Fig J, we can see that the parameter sensitivity ranking according to BP1 is $|\gamma_{k_4}| > |\gamma_{k_1}| > |\gamma_{k_2}|$, while the ranking based on BP2's change is $|\gamma_{k_1}| > |\gamma_{k_4}| > |\gamma_{k_2}|$. These results agree with our theoretical analysis (Eqn. (9)-(11)).
Then we take $k_3 = 0.62$ $k_3 = 0.8$ and $k_3 = 1.1$ to perform the parameter sensitivity analysis according to the change of barrier heights respectively. ΔV_1 is the barrier height from S1 to S2 (as shown in Fig K(a)), which can be obtained by computing Eqn. (12) and ΔV_2 is the barrier height from S2 to S1. The parameter sensitivity ranking of different k_3' give the same relation: $|V1_{k_4}| > |V1_{k_1}| > |V1_{k_2}|$according to the change of $\Delta V_1$and$|V2_{k_1}| > |V2_{k_4}| > |V2_{k_2}|$ according to the change of ΔV_2. Here we only show the results when $k_3 = 0.8$ as an example.

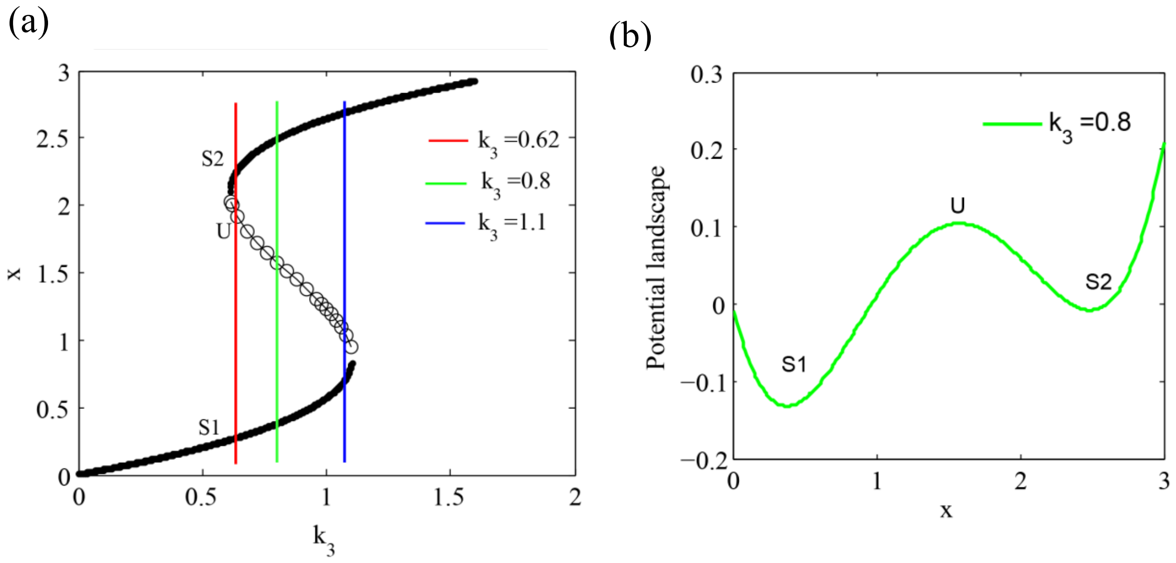

(c)

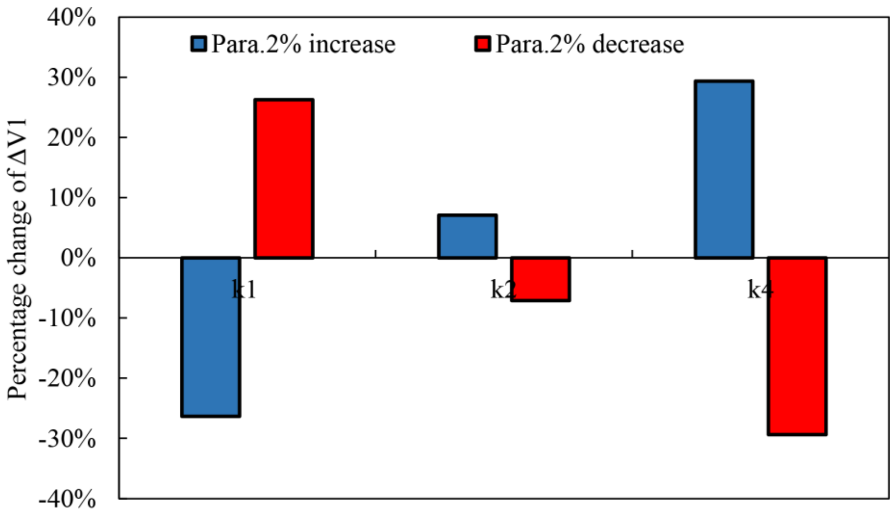

(d)

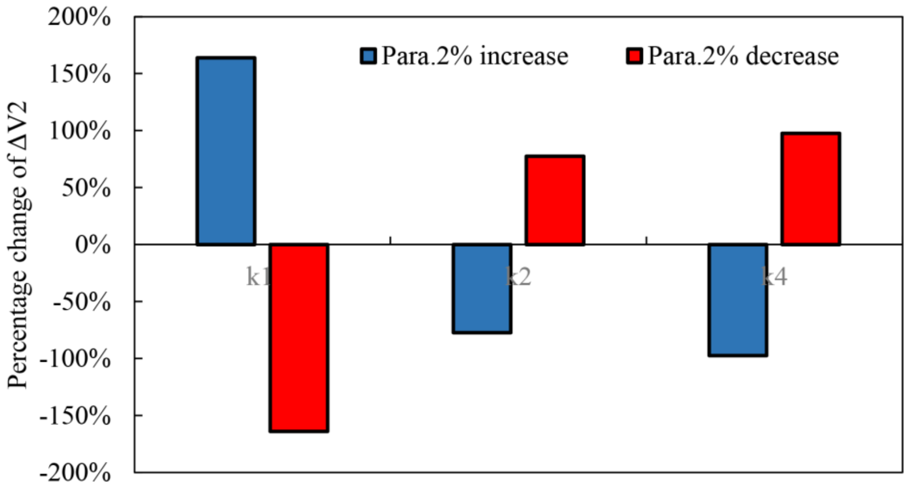

Fig K. (a) With a given k_3, there are three fix points of x including two stable points(S1 and S2) and one unstable fix point(U). (b)The potential landscape when $k_3 = 0.8$. (c) 2% decrease and increase in each parameter induced a percentage change of the barrier height ΔV_1. (d) 2% decrease and increase in each parameter induced a percentage change of the barrier height ΔV_2.

The parameter sensitivity ranking according to barrier height ΔV_1 is consistent with the ranking result according to BP1. Similar results hold for BP2. We also choose 3 more sets of parameters to repeat the above analysis. The equivalence for the parameter sensitivity obtained by deterministic bifurcation-point position analysis and barrier height analysis is again verified.

SI Reference

1. Albeck J G, Burke J M, Spencer S L, Lauffenburger DA, Sorger PK. Modeling a Snap-Action, Variable-Delay Switch Controlling Extrinsic Cell Death. Plos Biology, 2008, 6(12):2831-52.
2. Chen C, Cui J, Lu H, Wang R, Zhang S, Shen P. Modeling of the role of a Bax-activation switch in the mitochondrial apoptosis decision. Biophysical Journal, 2007, 92(12):4304-15.
3. Ku B, Liang C, Jung J U, Oh BH. Evidence that inhibition of BAX activation by BCL-2 involves its tight and preferential interaction with the BH3 domain of BAX. Cell Research, 2011, 21(4):627-41.
4. Zhou P, Li T. Construction of the landscape for multi-stable systems: Potential landscape, quasi-potential, A-type integral and beyond. Journal of Chemical Physics, 2016, 144(9):094109.
5. Freidlin MI, Wentzell AD. Random perturbations of dynamical systems, 2nd edition. Springer, 1998, New York.
6. Zi Z. Sensitivity analysis approaches applied to systems biology models. Iet Systems Biology, 2011, 5(6):336-6.
7. Bokov P M. Asymptotic Analysis for the Variance-Based Global Sensitivity Indices [J]. Science and Technology and Nuclear Installations, 2012,(2012-9-19), 2012, 2012(9):1-8.
8. Saltelli A., Ratto M., Andres T., Campolongo F., Cariboni J., Gatelli D., et al. "Global Sensitivity Analysis: The Primer", John Wiley & Sons, 2008
9. Schlögl, F. 1972 Chemical reaction models for nonequilibrium phase transition. Z. Physik. 253, 147–161.
